# The still valid fluid mosaic model for molecular organization of biomembranes: accumulating data confirm it

**DOI:** 10.15190/d.2013.7

**Published:** 2013-12-31

**Authors:** Mircea Leabu

**Affiliations:** University of Medicine and Pharmacy "Carol Davila", Department of Cellular and Molecular Medicine, Bucharest, Romania; "Victor Babes" National Institute of Pathology, Bucharest, Romania; University of Bucharest, Research Center for Applied Ethics, Bucharest, Romania

**Keywords:** membrane organization, membrane fluidity, membrane microdomains, Singer-Nicolson model, science history

## Abstract

More than forty years passed since Singer and Nicolson launched the fluid mosaic model related to molecular organization and dynamics of cell membranes, applicable to endomembranes as well. During this period of time, that will reach half a century soon, accumulating data all confirm, but not infirm the brilliant idea of such a model. Sometimes, the results developed the model in a very impacting manner, as was the case with the introduction of the membrane microdomain concept (mainly lipid rafts organization). From a didactical point of view, membrane microdomain organization suggests the mosaic’s “bricks” are even more complex than mere proteins or protein aggregates (the initial ones determining the parents of the model to design it). Current times, with high resolution equipments and techniques allowing live cell investigation, have opened new approaches resulting in enhancement of our understanding about biomembranes organization, dynamics and functioning. This paper will analyze some of the most recent data about membrane molecular organization and dynamics of biomembrane components, as well as interpretation of these data to see if they could modify the concept related to the fluid mosaic model. In a text assessment specific to papers in soft sciences, I will show the anticipatory and wise presentation of the fluid mosaic model by Singer and Nicolson, which has made it as a still valid one.

## Introduction

For 20 years I and perhaps many other professors have been teaching students in medicine about the molecular organization of the cell membrane, presenting with a high enthusiasm the fluid mosaic model launched by Seymour Jonathan Singer and Garth L. Nicolson in 1972^1^, and considering it as an inspired, still actual scientific and even pedagogic idea. A recent article by Akihiro Kusumi et al.^2^ proposed that the current advances on plasma membrane structure are critical and distinguish the cellular membrane structure from the model proposed by Singer and Nicolson in 1972. Although the manuscript is an important contribution in the field of membrane organization and functioning, some aspects and conclusions need to be further discussed and analyzed. Moreover, this paper follows a style specific to the contributions in soft sciences, namely a phrase assessment and comparisons with the affirmations of Singer and Nicolson published in their paper in Science, over forty years ago, and, for a stronger argumentation, in two other papers published by the parents of the model one year before, and by Singer alone two years after^3,4^. What’s the story?

The bewilderment that determined this paper started even by reading the abstract of the mentioned paper, where the authors admonished the readers that “The recent rapid accumulation of knowledge on the dynamics and structure of the plasma membrane has prompted major modifications of the textbook fluid-mosaic model.” Further on, as conclusions, in the abstract the authors have mentioned: “We propose that the cooperative action of the hierarchical three-tiered mesoscale (2–300 nm) domains – actin-membrane-skeleton induced compartments (40–300 nm), raft domains (2–20 nm), and dynamic protein complex domains (3–10 nm) – is critical for membrane function and distinguishes the plasma membrane from a classical Singer-Nicolson-type model.” After reading the paper entirely, no reason was found to consider the mentioned contributions very different from the fluid mosaic model as introduced and argued, in a very precautious and clever manner, by Singer and Nicolson. Furthermore, it was necessary to proceed at rereading the original papers, to refresh my memory and find reasons that my understanding of the fluid mosaic model does not result from my generosity, but on account of the argumentation that the parents of the model stated. The results of my research on the texts will be presented here.

## The textbook fluid mosaic model is an oversimplified one

**Figure 1:**
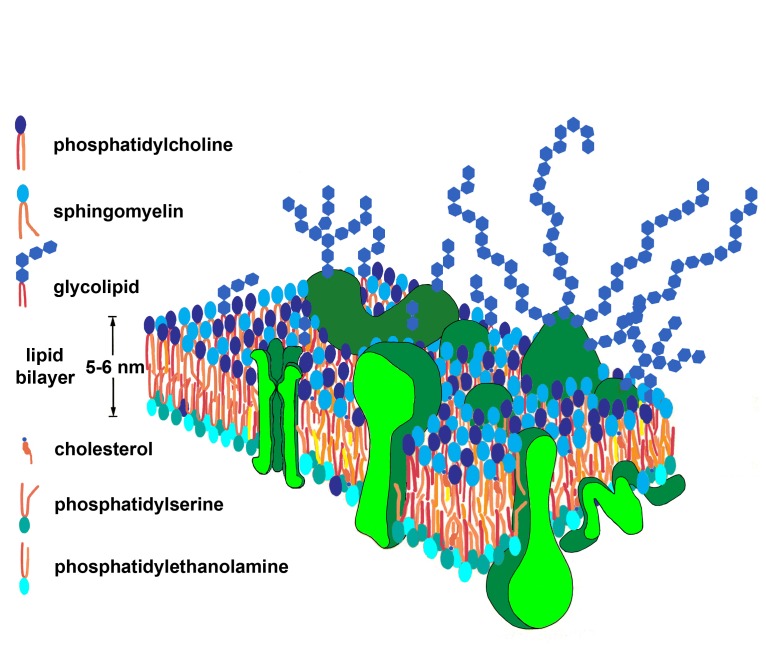
Fluid-Mosaic Model Image with an oversimplified presentation of the fluid mosaic model in textbooks. Although the heterogeneity and asymmetry in organization is obvious, no dynamics is shown in the image, no protein-protein association, no preferential interaction between lipids and proteins, and no specific organization of peripheral proteins on the cytoplasmic side, all modeling and/or restricting the movement of molecular components. Teachers have to take the responsibility to avoid misunderstanding of the model by the students.

The oversimplified presentation of the Singer and Nicolson model inside textbooks (Figure 1) stipulates that the basic element in membranes’ organization is a lipid bilayer, organized by amphiphilic membrane lipids, decorated with mosaics built by large protein macromolecules (floating on one or the other surface of, or immersed into the bilayer), with an oligo/poly-saccharide “cream” on the external side, carried by glycoconjugates (either glycolipids or glycoproteins/proteoglycans). The cohesiveness of these diverse molecular components – delimiting a cell by an ultrastructure without free ends – is maintained by noncovalent interactions, allowing a permanent movement of biocompounds in the membrane plane. This permanent moving of molecular components induces a two-dimensional fluidic behavior to the ultrastructure. Indeed, this simplified description of the fluid mosaic model could result in misunderstandings, but the eventual confusions are acceptable for beginners like students and not for scientists, a fact pointed out as a common occurrence by the authors of the review under discussion: “Many colleagues in other areas of biomedical science still consider the plasma membrane as a simple sea of lipids or, worse, as a solid plate, and they describe it as such in major textbooks, reviews, and research papers. Meanwhile, physicists and physical chemists still naively think that the molecular dynamics and interactions in the plasma membrane can be understood by directly applying the knowledge gained by using liposomes and artificially constructed simple membranes.” (See ref.^2^, p. 217) There is the teacher’s duty to avoid misunderstanding of the model by the students, on the one hand, and the responsibility of every scientist to carefully read papers and appropriately perceive the matter, on the other hand. No misunderstanding could find its source in Singer’s and Nicolson’s papers, published at the time, as I will try to prove below.

## The fluid mosaic model is a general one, but not necessarily universal

The review’s authors mention on page 217: “consider that virtually all biological membranes on Earth share the common (although peculiar) basic structure of a two-dimensional liquid, consisting of the mosaic-like mixture of lipids and proteins that undergo thermal diffusion in the two-dimensional space (Singer & Nicolson 1972), and that such universality and apparent uniqueness are comparable with those of the double-helical structure of DNA.” Here, the authors over interpreted Singer’s and Nicolson’s texts. The only reference to nucleic acids in their 1972 paper is related to explanations on scientific sources of the model they propose: “The fluid mosaic model has evolved by a series of stages from earlier versions […]. Thermodynamic considerations about membranes and membrane components initiated, and are still central to, these developments. These considerations derived from two decades of intensive studies of protein and nucleic acid structures; the thermodynamic principles involved, however, are perfectly general and apply to any macromolecular system in an aqueous environment. These principles and their application to membrane systems have been examined in detail elsewhere […] and are only summarized here.” (See ref.^1^, p. 720, under section “Thermodynamics and Membrane Structure”.) Moreover, a few paragraphs above, on the same page, Singer and Nicolson pointed out: “In particular, the mosaic appears to be a fluid or dynamic one and, for many purposes, is best thought of as a two-dimensional oriented viscous solution. In this article, we therefore present and discuss a fluid mosaic model of membrane structure, and propose that it is applicable to most biological membranes, such as plasmalemmal and intracellular membranes, including the membranes of different cell organelles, such as mitochondria and chloroplasts. These membranes are henceforth referred to as functional membranes. There may be some other membrane-like systems, such as myelin, or the lipoprotein membranes of small animal viruses, which we suggest may be rigid, rather than fluid, mosaic structures, but such membrane systems are not a primary concern of this article.” My question is: where is the “universality and apparent uniqueness […] comparable with those of the double-helical structure of DNA”?

## Fluidity in the mosaic model is not grounded in a Brownian movement of molecular components

The idea of a Brownian movement isn’t present anywhere in the three mentioned papers^1,3,4^ of Singer (two of them with Nicolson as co-author). Moreover, Singer and Nicolson have mentioned in their paper in 1971 that “some of the lipid may not be in the bulk bilayer phase, but may be more strongly interacting with proteins in the membrane” (see ref.^3^, p. 429). That means parents of the fluid mosaic model rather anticipated the sequestration of some lipids around transmembrane domains of the proteins, assuring them a right functional conformation, and accompanying them during their translational movement. The idea that the motility of membrane components follows other rules but not exactly a Brownian movement, because of putative interactions in-between, is largely expressed in the paper in Science: “The mosaic structure can be readily diversified in several ways. Although the nature of this diversification is a matter of speculation, it is important to recognize that the mosaic structure need not be restricted by the schematic representation in Fig. 2. [In Fig. 2 of their paper Singer and Nicolson showed only individual proteins floating, immersed or not, in/on the lipid bilayer.] Protein-protein interactions that are not explicitly considered in Fig. 2 may be important in determining the properties of the membrane. Such interactions may result either in the specific binding of a peripheral protein to the exterior exposed surface of a particular integral protein or in the association of two or more integral protein subunits to form a specific aggregate within the membrane. These features can be accommodated in Fig. 2 without any changes in the basic structure.” (See ref.^2^, p. 723) A few rows below, it is mentioned: “As has been discussed, however, a small portion of the lipid may be more intimately associated with the integral proteins.” Now, the review’s authors mentioned, benefiting from actual data, that “phospholipids, even those located in the outer leaflet of the plasma membrane, unexpectedly undergo hop diffusion between compartments with sizes similar to those detected by protein hop diffusion”, confirming Singer’s and Nicolson’s “premonition”. The question here is: what is so unexpected? I guess the unexpected was expected, but not experimentally proved for a long period of time. Other passages in Singer’s texts (as a unique author or with Nicolson as co-author) prove that they never considered the motility of membrane lipids and proteins as one completely free. They mentioned: “If indeed a plasma membrane is a two-dimensional solution of proteins in a viscous but fluid lipid solvent, then proteins and other membrane constituents not otherwise immobilized may be redistributed in the membrane when the membrane is subjected to any of a variety of chemical and physical perturbations.” (See ref.^3^, p. 433) or “Although membrane fluidity and lateral mobility of membrane components appear to be general and functionally important phenomena there is clear evidence that fluidity or mobility is restricted in certain membranes, or in regions of membranes, under particular conditions.” (See ref.^4^, p. 821) These statements speak for themselves.

## Molecular movement in the fluid mosaic model is modulated and restricted

Therefore, even the phrases quoted before confirm that Singer and Nicolson considered the membrane fluidity as a highly modulated feature, with several restrictions. Some of these restrictions are pointed out in their papers, as I will show below.

In their review, Kusumi and co-authors comment about the lower mobility of the membrane components showing experimental data proving it. They mention that “results clearly show that the 20-fold decrease in the diffusion coefficient in the plasma membrane, as compared with that in the simple Singer-Nicolson-type membrane (such as artificial reconstituted membranes, liposomal membranes, and the blebbed plasma membranes), is due to the influence of the actin filaments on the cytoplasmic surface of the plasma membrane. The data also clearly indicate that any theory for predicting the diffusion coefficient in the plasma membrane must include the effect of the actin filaments and that the application of most previous theories must be limited to only simple Singer-Nicolson-type membranes, such as artificial reconstituted membranes, liposomal membranes, and the blebbed plasma membranes […].”

What can we find on this issue in the pioneering papers of Singer and Nicolson? In their paper published in Science they mentioned: “The integral proteins would be expected to undergo translational diffusion within the membrane, at rates determined in part by the effective viscosity of the lipid, unless they were tied down by some specific interactions intrinsic or extrinsic to the membrane.” (See ref.^1^, p. 724)

Even though at the time the fluid mosaic model was introduced, the erythrocyte membrane was almost the only one studied (except the mitochondrion and chloroplast membranes), but definitely the best known in terms of biochemistry, Singer anticipated the role of spectrin in restricting lateral diffusion of other membrane proteins^4^. I’m extracting from Singer’s review paper, published in 1974 some suggestive passages arguing my affirmation in the previous sentence: “A related role proposed for the spectrin complex in erythrocytes is in controlling the lateral mobility of components in the erythrocyte membrane.” or “The capacity to be clustered must reflect the translational mobility of certain components in the plane of the membrane, and the results quoted show that this mobility is rather finely regulated in the erythrocyte membrane. It appears that this mobility can be markedly altered without a change in lipid composition or fluidity.” and “Some aggregation-disaggregation equilibrium of the spectrin complex itself might thereby control the lateral mobility of the intramembranous particle, but other integral proteins also might not diffuse as readily through this superstructure.” (See ref.^4^, p. 814 and next one) Further on, Singer made a generalizing suggestion: “I suggest that peripheral proteins will generally be attached to membranes by binding to the exposed hydrophilic ends of specific amphipathic integral proteins of the membrane.” He further mentioned that peripheral proteins’ “relatively weak binding to specific integral proteins in the membrane is their special feature, which serves to modulate and regulate specific membrane functions.” (See ref.^4^, p. 816) In the same paper, under the section titled “Membrane fluidity” (pp.820-822) ample passages can be found that prove Singer’s visionary opinions about the motility of membrane proteins. I will mention them: “Although membrane fluidity and lateral mobility of membrane components appear to be general and functionally important phenomena there is clear evidence that fluidity or mobility is restricted in certain membranes, or in regions of membranes, under particular conditions. Attention is therefore being directed to understanding the nature and mechanisms of such restrictions.” Another possibility could be that mentioned a few phrases later: “There are certain regions of specialized and ordered structure within eukaryotic cell membranes that are well recognized, such as synapses […] and gap junctions […]. […] In other words, it is a mosaic structure with the protein functioning as the rigid matrix, by contrast to a mosaic with a fluid lipid as the matrix; these could exist side by side in the same membrane.” Trying to reach a conclusion in regards to the diversity of information accumulated at the time, Singer pointed out: “None of this information is therefore irreconcilable with a fluid mosaic model for the usual functional membranes, although this is sometimes disputed.” In the same section other developments arguing the author’s large view on membrane components assembly and motility can be found: “In a previous section, the peripheral complex spectrin in erythrocyte membranes and its possible role in inhibiting the translational mobility of integral membrane components have been discussed, and it was suggested that similar actomyosin-like systems may operate with other cells and their membranes, perhaps at only certain times in the cell cycle […] and on specialized regions of the membrane” (see ref.^4^, p. 821). Moreover, commenting some contradictory reported results Singer pointed out that “the apparently contradictory findings that concanavalin A binding to normal 3T3 cells does […] and does not […] cause a clustering of their membrane receptors may be associated with the attachment of an actomyosin-like system to the plasma membrane in the latter case (in which cells attached to a substrate were examined), but not in the former (in which free cells suspension were used).” It is well known today that the cortical actin cytoskeleton is organized differently in the same cell, if it is attached or in suspension. Remaining open-minded, the author specified: “In other cases, other types of peripheral attachments may inhibit the mobility of integral components in the membrane.”

A last issue claimed by Kusumi and co-authors as a current contribution, to my understanding, is presented in the following passage: “Therefore, we propose that a paradigm shift for the long-range (>10 nm) structure of the plasma membrane is required: from the two-dimensional continuum fluid model of Singer and Nicolson to the compartmentalized fluid model, in which membrane molecules undergo short-term confined diffusion within a compartment and long-term hop diffusion between the compartments. The partitioning of the plasma membrane (compartments) by fences and pickets, which results in the observed hop diffusion of membrane molecules, makes the plasma membrane distinct from the simple Singer-Nicolson membrane. Thus, we consider the compartmentalization of the plasma membrane to be the first tier of the hierarchical mesodomain architecture of the plasma membrane.” Even for such an idea we can find commentaries in the paper by Singer and Nicolson, in Science. Let’s remember one after another the passages that could be related to this topic:

a. “There should generally be no long-range order in a mosaic membrane with a lipid matrix. By long-range, we mean over distances of the order of a few tenths of a micrometer and greater.”

b. “The absence of long-range order should not be taken to imply an absence of short-range order in the membrane. It is very likely that such short-range order does exist, as, for example, among at least some components of the electron transport chain in the mitochondrial inner membrane. Such short-range order is probably mediated by specific protein (and perhaps protein-lipid) interactions leading to the formation of stoichiometrically defined aggregates within the membrane. However, in a mosaic membrane with a lipid matrix, the long-range distribution of such aggregates would be expected to be random over the entire surface of the membrane.”

c. “The objection may immediately be raised that long-range order clearly exists in certain cases where differentiated structures (for example, synapses) are found within a membrane. We suggest, in such special cases, either that short-range specific interactions among integral proteins result in the formation of an unusually large two-dimensional aggregate or that some agent extrinsic to the membrane (either inside or outside the cell) interacts multiply with specific integral proteins to produce a clustering of those proteins in a limited area of the membrane surface. In other words, we suggest that long-range random arrangements in membranes are the norm; wherever nonrandom distributions are found, mechanisms must exist which are responsible for them.”

d. “The integral proteins would be expected to undergo translational diffusion within the membrane, at rates determined in part by the effective viscosity of the lipid, unless they were tied down by some specific interactions intrinsic or extrinsic to the membrane.”

It seems obvious to me that no fundamental contradiction could be identified between Singer’s and Nicolson’s points of view in 1972 and developments brought by the review of Kusumi *et al*. No doubt, the “three-tiered hierarchical dynamic organization of molecules in the plasma membrane” is a deserving contribution to our understanding of membrane molecular organization and functioning, but no distinguishable feature contradicting the original fluid mosaic model could be extracted. The contribution of the review by Kusumi *et al*. is an important detailed description, with a high impact on our knowledge, but does not cancel anything in the Singer and Nicolson model. It is an additional contribution, as was the introduction of membrane microdomains (with the classical example of lipid rafts^5-8^).

## Changing unanimous accepted opinions, but not the fluid mosaic model concept

I use to talk to my students about the ingenuity of the cell in finding solutions for every unpredictable, impossible or unreasonable issue (based on our knowledge at a given time). Moreover, to practice my ludic spirit, I may point out that in the Romanian language the word “cell” is feminine in genre, so we may expect “her” to be unpredictable, even capricious, but very practical.

In the context of our assessment, two issues will be pointed out related to facts changing the unanimous accepted opinions after experimental data proved them to be incorrect.

The first one is about the conformation of transmembrane domains of integral proteins completely passing the bilayer. Even Singer and Nicolson mentioned in their initial papers that “proteins of a variety of intact membranes, on the average, show appreciable amounts of the α-helical conformation” (see ref.^1^, p. 722). For a long period of time, the packaging as an α-helix of the transmembrane domains of integral membrane proteins was considered as the only possibility. That was supported by thermodynamic reasons. The polypeptide chain arranged in an α-helix conformation assure the hydrophilic moieties to be hidden toward the helix axis, while hydrophobic parts of the protein are oriented toward the lipids of the bilayer, accommodating one-another. Everybody agreed on this point. Moreover, transmembrane domains of integral proteins contain mainly hydrophobic amino acids. In his review in 1974, Singer detailed the idea, talking about glycophorin: “there is an intervening sequence of about 23 amino acid residues which are predominantly hydrophobic. It is suggested that if this stretch of residues was arranged in a continuous α-helix, it would be long enough to span the membrane.” (See ref. ^4^, p. 811) However, in 1978 Stephen J. Kennedy^9^ introduced the idea of polypeptide chain organization as a barrel, whose staves are β-pleated sheets, a structure that could form the transmembrane domains of some integral proteins. Later, such an organization of transmembrane domains was proved for porins – transmembrane proteins in the outer membrane of some bacteria and the outer membrane of mitochondria. Singer himself mentioned this new vision about organization of transmembrane domains for some integral proteins in one of his later reviews^10^. Therefore, the cell did that, but we had to accept its ingenuity.

The second issue is the controversy related to the existence of integral proteins partially immersed in the bilayer, which means integral proteins that are not transmembrane proteins. Again, argumentation claims thermodynamics: it seems almost impossible for a polypeptide chain to return inside the membrane, following a hairpin trajectory, without exposing hydrophilic residues toward the hydrophobic part of the bilayer. The debate was long lasting, despite the fact that in his review in 1974, Singer considered data related to cytochrome b_5_ and cytochrome b_5_ reductase suggesting they are integral proteins partially immersed into the bilayer (see ref.^4^, p. 810, 812). The facts became more acceptable after caveolin investigation, the protein organizing caveolae. For caveolin, a credible model for polypeptide chain topological organization was presented in 2005^11^. Again, the cell found solution for its needs, and we had to find explanations by designing models.

None of the two proofs – that determined the scientific community to change its opinions – contradicts the fluid mosaic model, but are accommodated by it.

## Concluding remarks

Kusumi and co-authors’ contribution is rather a development of the fluid mosaic model for membrane molecular organization and for membrane components behavior, but not a distinguished alternative. If people misunderstood the fluid mosaic model, due to the oversimplified presentation in textbooks, Singer and Nicolson cannot be held accountable for it.

The contribution by Akihiro Kusumi *et al*.^2^ is definitely an extremely important one. Many professors will probably use the reviewed information in this paper in their teaching activity, promoting the gained knowledge. Beyond being a polemic, this current paper tries to give its due to the science history related to the development of our knowledge about molecular organization and dynamics of biomembranes. Fortunately, other authors do the same^5,6^. Therefore, in his anniversary paper, Michel Morange pointed out: “The unexpected resistance of the lipid bilayer model, as well as new data demonstrating the rapid displacement of proteins within the membrane plane, led to a progressively elaborated synthesis that was brilliantly outlined by Singer and Nicolson in their famous Science article.” Moreover, he mentioned that the integrative work of the two scientists had a positive destiny: “The new model was rapidly and unanimously accepted, and it remained unaltered during the next forty years.”

Scientific knowledge has usually advanced by data accumulation and building new concepts or paradigms by an integrative work, without any demolition of the older gains. Sometimes, changes need to destroy mentalities and older concepts to advance the knowledge. It is not (yet?!) the case with the Singer’s and Nicolson’s fluid mosaic model for biomembranes. Let’s stay open-minded.

- **The Singer's and Nicolson's fluid-mosaic model released in 1972 is still valid**, **being confirmed and refined by all further discoveries.**
